# Internal conceptual replications do not increase independent replication success

**DOI:** 10.3758/s13423-016-1030-9

**Published:** 2016-04-11

**Authors:** Richard Kunert

**Affiliations:** 1 Max-Planck-Institut für Psycholinguistik, Wundtlaan 1, 6525 XD Nijmegen, Netherlands; 2 Donders Institute for Brain, Cognition and Behaviour, Radboud University, Kapittelweg 29, 6525 EN Nijmegen, Netherlands

**Keywords:** Replication, Reproducibility, QRP, False positives, Publication bias

## Abstract

Recently, many psychological effects have been surprisingly difficult to reproduce. This article asks why, and investigates whether conceptually replicating an effect in the original publication is related to the success of independent, direct replications. Two prominent accounts of low reproducibility make different predictions in this respect. One account suggests that psychological phenomena are dependent on unknown contexts that are not reproduced in independent replication attempts. By this account, internal replications indicate that a finding is more robust and, thus, that it is easier to independently replicate it. An alternative account suggests that researchers employ questionable research practices (QRPs), which increase false positive rates. By this account, the success of internal replications may just be the result of QRPs and, thus, internal replications are not predictive of independent replication success. The data of a large reproducibility project support the QRP account: replicating an effect in the original publication is not related to independent replication success. Additional analyses reveal that internally replicated and internally unreplicated effects are not very different in terms of variables associated with replication success. Moreover, social psychological effects in particular appear to lack any benefit from internal replications. Overall, these results indicate that, in this dataset at least, the influence of QRPs is at the heart of failures to replicate psychological findings, especially in social psychology. Variable, unknown contexts appear to play only a relatively minor role. I recommend practical solutions for how QRPs can be avoided.

**Electronic supplementary material:**

The online version of this article (doi:10.3758/s13423-016-1030-9) contains supplementary material, which is available to authorized users.

## Introduction

The hallmark of scientific evidence is its reproducibility. Recently, the Open Science Collaboration (2015) found that psychological science is less reproducible than desired. This reproducibility project tried to independently replicate 100 effects, of which 97 were statistically significant in the original publications. Even though an estimated average power of 92 % for replication experiments would predict 89 successful replications, only 35 were observed. Moreover, 82 out of 99 studies for which effect sizes could be calculated showed smaller replication effect sizes than original estimates. This paper asks a simple question: are internal replications, i.e. showing an effect more than once in a given publication, predictive of independent replication success? The answer to this question can contribute to our understanding of why many independent replications were unsuccessful, and what can be done in order to avoid low replication rates in the future.

According to the unknown moderator account of independent replication failure, successful internal replications should correlate with independent replication success. This account suggests that replication failure is due to the fact that psychological phenomena are highly context-dependent, and replicating seemingly irrelevant contexts (i.e. unknown moderators) is rare (e.g., Barrett, 2015; DGPS, 2015; Fleming Crim, 2015; see also Stroebe & Strack, 2014; for a critique, see Simons, 2014). For example, some psychological phenomenon may unknowingly be dependent on time of day. Data acquisition in the morning reveals it while in the afternoon the effect is absent. The unknown moderator account predicts that successful internal replications (which were overwhelmingly conceptual replications) increase independent (direct) replication success because an internally replicated phenomenon is less likely to be a chance finding, and more likely to be found despite small variations in experimental design, compared to a phenomenon without internal replication.

The latter point rests on the distinction between conceptual and direct replications, represented here by internal and independent replications, respectively. Conceptual replications test the same theory with variable experimental designs. Internal replications were overwhelmingly of this type. In contrast, direct replications attempt to recreate an experimental design as closely as possible. Independent replications were of this type because replication teams consulted with original authors and used original materials in order to minimize procedural differences between original and independent replication studies (Open Science Collaboration, 2015). Therefore, procedural differences between studies, which the unknown moderator account invokes in order to explain replication failures, were *intended* for internal, conceptual replications. Thus, if a phenomenon can be reproduced with intentionally more procedural differences (internal, conceptual replications) it should be possible to reproduce it also with fewer procedural differences (independent, direct replications).

Of course, for a single pair of original and replication studies, the *kind* of procedural differences is important rather than their number. However, for a collection of original-replication pairs, the greater the number of procedural differences between original and replication studies, the greater the chances that some differences of importance (e.g. crucial replication contexts) are among them. This chance is greater for internal, conceptual replications than for independent, direct replications. Hence, according to the unknown moderator account, the existence of successful internal replications predicts that a psychological phenomenon is more robust against small variations in experimental design and, hence, that independent replications will be successful.

A second account of independent replication failure predicts no independent replication difference between effects with or without internal replications. This account attributes low independent replication success to the fact that questionable research practices (QRPs) employed in original studies were not applied during replication attempts. Examples of QRPs are (for a longer list, see Asendorpf et al., 2013):

1. Optional stopping (‘sampling until significant’)
  A researcher repeatedly tests the data during acquisition and stops sampling once the *P*-value is below .05. This is not an uncommon practice as revealed by the 5 %–23 % of surveyed psychological researchers admitting to having stopped sampling early, and 32 %–58 % admitting to having stopped late based on the results (Fiedler & Schwarz, 2015; John, Loewenstein, & Prelec, 2012). In practice, this QRP can increase the false positive rate to 22 %–29 % (Simmons, Nelson, & Simonsohn, 2011; data simulations in Supplementary materials), while in theory even 100 % false positives are possible (Wagenmakers, 2007).
2. Publication bias (the ‘file drawer’ problem; Rosenthal, 1979)
  Researchers are reluctant to write up non-significant results, as revealed by the fate of preregistered studies in the social sciences in general (Franco, Malhotra, & Simonovits, 2014), and in psychology in particular (Franco, Malhotra, & Simonovits, 2016). Survey results are in line with these findings: 42 %–50 % of psychological researchers admit to at least once having only reported studies that “worked” (Fiedler & Schwarz, 2015; John et al., 2012). Moreover, it is commonly believed that scientific journals are reluctant to publish non-significant results. Both kinds of bias result in publication bias, the tendency is for significant results to be published while non-significant results remain unpublished (see also LeBel et al., 2013).
3. HARKing (hypothesizing after a result is known; Kerr, 1998)
  All effects are reported as supporting the hypotheses. If an effect happens to be in an unexpected direction, the hypothesis is adjusted *post hoc* to make it seem as if the direction of the effect was expected after all, i.e. effect sizes are never negative (de Groot, 1956/2014). A common practice that 35 %–45 % of surveyed psychological researchers admit to (Fiedler & Schwarz, 2015; John et al., 2012).

Data simulations have repeatedly shown that QRPs reduce research effort, e.g., in terms of lowering the sample size per study, while increasing the false positive rate and exaggerating the estimated effect size (Bakker, Dijk, & Wicherts, 2012; Guan & Vandekerckhove, 2015; Simmons et al., 2011; see also data simulations in Supplementary materials). Therefore, if a researcher wants to claim that a new finding is replicable, s/he can simply run several studies, employing QRPs in each case and risking more than one false-positive finding. As a result, the QRP account predicts that internal replications, i.e. showing an effect more than once in the same publication, are not predictive of independent replication success (for a different approach which also uses the existence of internal replications for arguing that QRPs were used, see Francis, 2014; Francis, Tanzman, & Matthews, 2014; Schimmack, 2012).

Overall, the difference between the two explanations lies in the fact that, under the unknown moderator account, original and replication studies tap into slightly different true effects (independent of research practices) while the QRP account attributes low replication rates to the practices themselves. Thus, did the Open Science Collaboration (2015) successfully reproduce internally replicated effects more often than internally unreplicated effects (prediction by unknown moderator account) or not (prediction by QRP account)? Here, I will re-analyze the data acquired by the Open Science Collaboration (2015) in order to address this question by examining predictions from both explanations for the low independent replication success.

## 1. Contrasting reproducibility between internally replicated effects and internally unreplicated effects

## Methods

### Data set

**Fig. 1:**
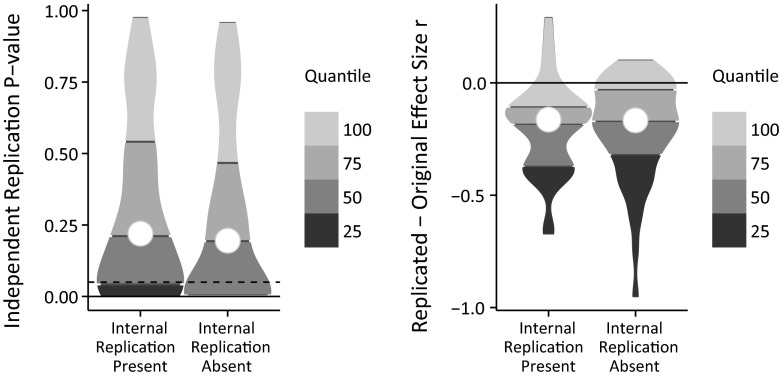
Comparing the reproducibility of internally replicated and unreplicated effects in an empirical dataset. *Left panel P*-values obtained by independent replication teams. The *dotted line* represents the threshold for considering an effect statistically significant (*P* = .05). Note that the bottom 25 % quartile in the right distribution of the *left panel* relating to previously internally unreplicated effects is at *P* = .0017 and, thus, not visible here. *Right panel* Reduction in effect size between original study and independent replication. *Violin plots* display density, i.e. thicker parts represent more data points

The reproducibility project’s dataset of 100 independent replication studies was used (for details, see Open Science Collaboration, 2015). Of the original effects, 44 were internally, conceptually replicated, 20 once, 10 twice, 9 three times, and 5 more than three times. Here, I simply contrast the reproducibility of internally replicated and internally unreplicated effects, see Fig. 1.^1^

### Analysis

R-code for re-creating all figures and analyses is provided in the Supplementary materials. In a first analysis I calculated the Bayes factor, which represents the relative evidence for one model over another: the null model of no difference between internally replicated and not internally replicated effects (QRP account), and the alternative model of greater replication success for internally replicated compared to not internally replicated effects (unknown moderator account). I used Morey, Rouder, & Jamil's (2015) BayesFactor package in R in order to compare proportions (contingency table Bayes factor test; Gunel & Dickey, 1974) and scores (Bayesian independent *t*-test; Rouder, Speckman, Sun, Morey, & Iverson, 2009). The latter analysis assumes a normal distribution. In case normality was not met and could not be reached through data transformations, the Bayes factor is reported only for completion.

I follow common practice for characterizing relative model support based on Bayes factors: BF_0+_ > 1 indicates support for the null hypothesis (QRP account), BF_+0_ > 1 indicates support for the alternative model (unknown moderator account). Jeffreys (1961) suggests that 1 < BF < 3 provides model evidence that is not worth more than a bare mention; 3 < BF < 10 indicates that the evidence for a hypothesis is substantial, when 10 < BF < 30 it is strong.

A second Bayesian analysis was performed using parameter estimation based on 100,000 samples from the posterior distribution (log odds ratio for contingency table, difference score for *t*-test). The estimated parameters are a formal representation of the belief in the difference between internally replicated and internally unreplicated effects. The 95 % Credible Interval is a measure of uncertainty about this belief. Please note that Bayesian estimation of difference scores used Krushke’s BEST package (Kruschke, 2013; Meredith & Kruschke, 2015), which does not assume normality. Therefore, data were not transformed and the estimated parameters are straightforward to interpret.

## Results

Figure 1 shows that there is no independent replication advantage for original studies that internally replicated an effect compared to those that did not, see Table 1 for formal analyses. The left panel of Fig. 1 does not indicate any support for the unknown moderator account that predicts lower independent replication *P*-values for internally replicated compared to internally unreplicated effects. The mean (white dot), median (middle dark grey line), and inter-quartile range (upper and lower dark grey lines) all show a difference in the unpredicted direction (*P*_internally unreplicated_ < *P*_internally replicated_).

**Table 1:** Comparison of internally replicated and internally unreplicated effects

	Internal replication present	Internal replication absent	Bayes factor	Posterior median [95 % Credible Interval]^a^
Reproducibility				
Independent replications *P* < .05	12 out of 42	22 out of 54	BF_0+_ = 8.72	−0.52
				[−1.39; 0.31]
Effect size reduction (simple subtraction)^b^	*M* = 0.20	*M* = 0.20	BF_0+_ = 4.15	−0.00
	(SD = 0.20)	(SD = 0.22)		[−0.09; 0.08]
Effect size reduction (Cohen’s *q*)^b^	*M* = 0.20	*M* = 0.24	BF_0+_ = 2.43	0.00
	(SD = 0.26)	(SD = 0.27)		[−0.10; 0.10]
Reproducibility predictors				
Field of study	13 × cognitive	29 × cognitive	BF_+0_ = 5.76	0.22
	29 × social	25 × social		[0.03; 0.40]
Effect type	20 × main effect	29 × main effect	BF_0+_ = 3.13	0.02
	16 × interaction	21 × interaction		[−0.18; 0.23]
Original study *P*-value^b^	*M* = .015	*M* = .013	BF_0+_ = 2.78	0.00
	(SD = .016)	(SD = .016)		[−0.00; 0.01]
Original effect size	*M* = .36	*M* = .42	BF_+0_ = 1.42	0.07
	(SD = .15)	(SD = .22)		[−0.01; 0.14]
Independent replication power^b^	*M* = .92	*M* = .92	BF_0+_ = 3.64	0.01
	(SD = .08)	(SD = .09)		[−0.02; 0.04]
Surprisingness of original effect^c^	*M* = 3.19	*M* = 2.97	BF_0+_ = 1.36	0.21
	(SD = 0.98)	(SD = 0.83)		[−0.17; 0.60]
Challenge of conducting replication^b,d^	*M* = −.06	*M* = −.05	BF_0+_ = 4.74	−0.03
	(SD = 0.79)	(SD = 0.82)		[−0.36; 0.31]
Formal power analysis in original publication present/absent	0 × present	2 × present	BF_0+_ = 22.21	−0.03
	42 × absent	52 × absent		[−0.11; 0.04]
Sample size of original study^e^	*M* = 71.00	*M* = 92.44	BF_0+_ = 4.41	−6.25
	(SD = 55.77)	(SD = 124.12)		[−25.94; 14.50]

^a^Positive values represent support for the alternative hypothesis representing the unknown moderator account

^b^Data not normally distributed. No satisfactory data transformation could be found. The reader should therefore focus on parameter estimation which does not assume normality

^c^Based on mean of three raters using Likert scale from 1 (not at all surprising) to 6 (extremely surprising)

^d^Based on combination of three standardized mean ratings as in Open Science Collaboration (2015)

^e^Natural logarithm of raw data due to non-normal distribution of raw values. Raw data results in BF_0+_ = 1.74. Analysis excludes one study with an unusual sample size (*N* = 230,025)

I use *P* < .05 as a measure of independent replication success (Fig. 1 left panel, dotted line) and compare replication success proportions using the Bayes factor and parameter estimation. The contingency table Bayes factor of BF_0+_ = 8.72 indicates substantial support for the null hypothesis of no difference (representing the QRP account) over the alternative hypothesis of a greater proportion of *P* < .05 for internally replicated effects (29 % replication success) compared to internally unreplicated effects (41 % replication success). Moreover, the posterior median of the log odds is negative at −0.52, counterintuitively implying that the presence of internal replications *reduces* the chances of independent replication success. However, the uncertainty about this reversed replication advantage is noteworthy [95 % Credible Interval (−1.39; 0.31)]. Overall, the comparison of independent replication *P*-values supports the QRP account that predicts no difference between internally replicated and internally unreplicated effects.

If the reduction in effect size between original and replication study is used as the criterion for replication success, the conclusion is the same. Looking at the right panel of Fig. 1 does not indicate any support for the unknown moderator account, which predicts an effect size reduction closer to zero for internally replicated effects (observed *M* = .20, SD = .20) compared to internally unreplicated effects (observed *M* = .20, SD = .22). Again the median and the interquartile range are in the opposite direction (*r*_difference_internally unreplicated_ closer to zero than *r*_difference_internally replicated_) of what the unknown moderator account predicts.

Given that the normality assumption is not met, I only discuss parameter estimation results, see Table 1. The posterior median of the difference between effect size reductions of previously internally replicated and previously internally unreplicated effects is zero. The 95 % Credible Interval is narrow, never even extending to a difference of anything else than trivial (trivial effects have values of |r| < .1; Cohen, 1992). The picture is very similar when following the practice of the Open Science Collaboration (2015) in using Fisher transformed effect sizes (Cohen’s *q*) for the same comparison (trivial differences have |*q*| < .1, Cohen, 1992). The formal analysis supports the aforementioned visual impression: the difference between original and replication effect sizes is practically the same whether an effect was internally replicated or not, as predicted by the QRP account.

## Discussion

Internal conceptual replications do not improve independent replication outcomes, as predicted by the QRP account. This finding is in line with an unrelated, recent Bayesian re-analysis of the reproducibility project’s dataset (Etz & Vandekerckhove, 2016). However, proponents of the unknown moderator account could argue that the presence of internal replications is just one of many factors predicting reproducibility. Do other reproducibility predictors counteract the influence of internal replications on independent reproducibility?

## 2. Contrasting reproducibility predictors between internally replicated and internally unreplicated effects

## Methods

### Data set

I use the same data set as above.

### Analysis

The Open Science Collaboration (2015) identified seven reproducibility predictors: field of study, effect type (main or interaction), original study *P*-value, original study effect size, replication power, surprisingness of the original effect, challenge of conducting the replication. I also include the presence of a formal power analysis and original sample size in this comparison based on the suggestion of a reviewer.

The formal analysis is along the lines seen above. The QRP account again predicts no difference between internally replicated and internally unreplicated effects in terms of reproducibility predictors (null hypothesis). The unknown moderator account predicts that factors favoring reproducibility are more common in internally unreplicated effects compared to internally replicated effects. This would explain why, under this account, the presence of internal replications—looked at in isolation—is not predictive of independent replication success.

## Results

In general, original studies with and without internal replications were very similar with respect to factors predicting reproducibility, see Table 1 (BF_0+_ > 3, posterior centred near zero). For some predictors, the evidence was inconclusive, see Table 1 (BF_0+_ < 3, BF_+0_ < 3, posterior not centred near zero but 95 % Credible Interval includes zero). There is one exception to this general pattern: the field of study (BF_+0_ = 5.76). Effects that were internally replicated were more likely to be classified as social psychological effects (69 %), while effects which were not internally replicated were mostly (54 %) cognitive effects. In other words, internal replications cannot fully remove the influence of the field of study (social psychological effects are difficult to replicate) on independent replication success.

This unexpected result raises the obvious question of whether the unknown moderator account is well supported in at least one field of study. However, this is not the case, see Table 2. The Bayes factors either support the null hypothesis (social psychology), or they are inconclusive (cognitive psychology). Parameter estimation of the difference in effect size reductions between internally replicated and internally unreplicated effects leads to a similar conclusion. In social psychology, the effect size reduction difference’s 95 % Credible Interval ranges from a small negative difference (the opposite to the unknown moderator account’s prediction) to a trivial positive difference (for both simple reduction and Cohen’s *q*). In cognitive psychology, on the other hand, the 95 % Credible Interval is nearly twice as big, ranging from small negative differences to small positive differences, i.e. the data do not support either hypothesis strongly.

**Table 2:** Comparison of internally replicated and internally unreplicated effects for different fields of study

	Internal replication present	Internal replication absent	Bayes factor	Posterior median [95 % Credible Interval]^a^
Social psychology				
Independent replications *P* < .05	5 out of 29	8 out of 25	BF_0+_ = 7.60	−0.76
				[−2.03; 0.45]
Effect size reduction (simple subtraction)^b^	*M* = 0.22	*M* = 0.17	BF_0+_ = 7.15	−0.06
	(*SD* = 0.16)	(*SD* = 0.17)		[−0.15; 0.04]
Effect size reduction (Cohen’s *q*)^b^	*M* = 0.23	*M* = 0.17	BF_0+_ = 6.96	−0.06
	(*SD* = 0.18)	(*SD* = 0.19)		[−0.17; 0.04]
Cognitive psychology				
Independent replications *P* < .05	7 out of 13	14 out of 29	BF_0+_ = 1.92	0.21
				[−1.05; 1.49]
Effect size reduction (simple subtraction)^b^	*M* = 0.15	*M* = 0.24	BF_0+_ = 1.26	0.08
	(*SD* = 0.26)	(*SD* = 0.25)		[−0.10; 0.26]
Effect size reduction (Cohen’s *q*)^b^	*M* = 0.13	*M* = 0.29	BF_+0_ = 1.38	0.13
	(*SD* = 0.36)	(*SD* = 0.32)		[−0.11; 0.38]

^a^Positive values represent support for the alternative hypothesis representing the unknown moderator account

^b^Data not normally distributed. No satisfactory data transformation could be found. The reader should therefore focus on parameter estimation which does not assume normality

## Discussion

Are factors favouring reproducibility more common in internally unreplicated effects compared to internally replicated effects, as predicted by the unknown moderator account? There is not much evidence for this proposal. While it is true that there is a difference between internally replicated and internally unreplicated effects in terms of field of study, neither field convincingly displays an independent replication advantage for internally replicated effects. Whether internally replicated and unreplicated effects differ on unknown variables predicting replication success is unclear, given that this analysis uses correlational data. Overall, in line with analysis 1, analysis 2 found support for the QRP account.

## General discussion

Why were many psychological effects not reproduced by the Open Science Collaboration (2015)? One account suggests that replication teams tapped into smaller, or even null, population effects because they did not re-create important experimental contexts (unknown moderator account). This account predicts that internal replications increase independent replication success. Another account suggests that original researchers used QRPs, which exaggerated their results, while the replication teams did not use them (QRP account). By this account, internal replications should not correlate with independent replication success. Given that internal replications are *not* predictive of independent replication success, the QRP account appears to be the better explanation, see Table 1. Moreover, the lack of predictive value of internal replications is not simply due to other reproducibility predictors counter-acting the influence of internal replications on independent replication success, see Section 2.

Still, a proponent of the unknown moderator account might argue that, as soon as the data analysis context changes, reproducibility cannot be achieved. For example, whether internally, conceptually replicating an effect in the morning or not, a direct, independent replication attempt in the afternoon will not show some phenomenon that is dependent on time of day. However, this argument misses two points. First, the influence of *unknown* moderators is not predictable, i.e. it is a process governed by random chance. When the chances of unknown moderator influences are greater and replicability is achieved (internal, conceptual replications), then the same should be true when chances are smaller (independent, direct replications). Second, the unknown moderator account is usually invoked for social psychological effects (e.g. Cesario, 2014; Stroebe & Strack, 2014). However, the lack of influence of internal replications on independent replication success is not limited to social psychology. Even for cognitive psychology a similar pattern appears to hold.

Could psychological findings be more replicable? The results are encouraging. Low reproducibility is not a feature of psychological science that derives exclusively from the allegedly variable, context-dependent nature of psychological phenomena. If differences in research strategy and investigated effects can be minimized, better reproducibility is possible. Firstly, the Open Science Collaboration has shown how to minimize the chances of investigating slightly different effects in original and replication studies. They consulted with original authors and used original materials (Open Science Collaboration, 2015).

Secondly, reproducibility can be boosted by avoiding QRPs. For example, optional stopping is not a QRP if statistical tests are appropriately adjusted (Lakens, 2014; Sanborn & Hills, 2013; Wagenmakers, 2007), publication bias can be avoided by promoting dedicated publication outlets open to unclear/null findings (e.g. PLoS ONE, prep-print servers, psychfiledrawer.org), hypothesizing after a result is known is prevented by basing hypotheses on earlier publications before sampling begins.

However, the wider challenge lies in removing the *incentives* for applying QRPs (for a list of suggestions, see Asendorpf et al., 2013; Ioannidis, Munafò, Fusar-Poli, Nosek, & Lakens, 2014; Kerr, 1998). Otherwise, human ingenuity will likely continue to find ways to present as reliable what is in truth irreproducible. One promising improvement lies in altering publication practices, encouraging a two-stage manuscript submission process that decouples editorial decisions from study results (e.g., pre-registration: Chambers, 2013; Greve, Bröder, & Erdfelder, 2013; Nosek & Lakens, 2014; or withholding results from reviewers: Smulders, 2013; Walster & Cleary, 1970). This report suggests that, without widespread changes to psychological science, it will become difficult to distinguish it from informal observations, anecdotes and guess work.

## Electronic supplementary material

Below is the link to the electronic supplementary material.

ESM 1 (PDF 143 kb)

ESM 2 (PDF 62 kb)

ESM 3 (PDF 14.8 kb)

ESM 4 (PDF 8.22 kb)

## Footnotes

1 Data for *P*-value comparison: study pairs with statistically significant original effects and exact replication *P*-values (*N* = 96, 44 % internally replicated). Data for effect size reduction comparison: studies whose effect sizes could be calculated (*N* = 97, 42 % internally replicated).
