# Supplementary material for: Internal conceptual replications do not increase independent replication success
Source: Psychon Bull Rev. 2016 Apr 11;23(5):1631–8. doi: 10.3758/s13423-016-1030-9 (PMC5050250; doi:10.3758/s13423-016-1030-9)
Supplement: Supplementary file 1 — (PDF 143 kb) [file 13423_2016_1030_MOESM1_ESM.pdf]

Supplementary Material

Questionable research practices increase chance findings and lead to overestimated effect sizes

I simulated a research team faced with the task of minimizing the number of experimental participants while maximizing the output of publishable studies which “worked” (statistically significant result). Simulations include a series of studies with a within-subjects design, two conditions, ten to 100 participants, and a known standardized mean difference between conditions (d_z). The correlation coefficient *r* will be used to report effect sizes (Open Science Collaboration, 2015).

Researchers can follow six strategies.

(1) They are not knowledgeable about human psychology and simply sample a random number of participants (sample size *N* is a random draw from a uniform distribution with minimum 10 and maximum 100), followed by a *t*-test on the data.

(2) They expect a certain effect size based on a careful reading of the psychological research literature. Using this effect size in a power-analysis gives a required sample size for obtaining a significant effect with 80% chance, should the effect really exist (80% power). They recruit as many participants as necessary for 80% power, followed by a *t*-test.

The team observes that some of their colleagues produce more research publications in higher impact journals which improve these colleagues’ chances for promotion, so they apply one or all of the QRPs outlined in the main text:

(3) Optional stopping

(4) Publication bias

(5) HARKing (hypothesizing after a result is known)

(6) In order to maximize the own success rate, the researchers combine all three questionable research practices.

I simulated the team investigating a range of psychological phenomena with population effect sizes between *r* = 0 (null effect) and *r* = .8 (large effect). The results of 15,000 investigations of each effect are summarized in Figure S1, beginning with the number of participants the researchers sampled (top left panel), the resulting proportion of studies which "worked" (top right panel), and the observed effect sizes (bottom left panel). Note that the x-axis represents the true, population effect sizes which are unknown in real life (and thus need to be investigated by researchers), while the y-axis (bottom left panel) represents the observed effect sizes derived from the samples. The simulations clearly show why the colleagues using QRPs were so successful (Figure S1: triangles): they spent less time acquiring data and had more successful studies with greater observed effect sizes compared to researchers not engaging in such practices (Figure S1: dots).

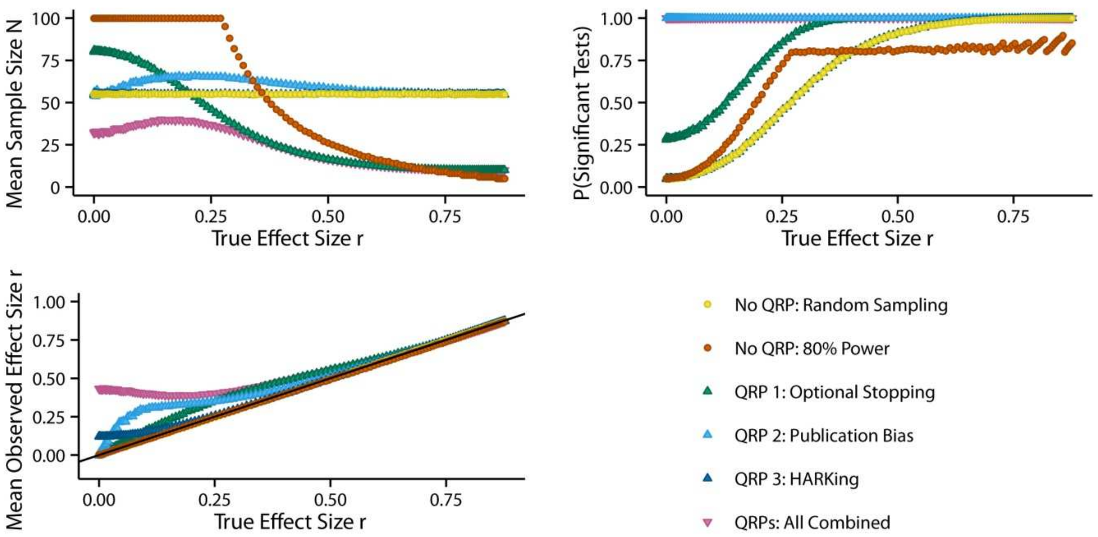

Figure S1. The effect of different research strategies on invested resources and observed results. The **top left panel** shows mean sample sizes at different sizes of an experimental effect for six different research strategies. The **top right panel** shows the proportion of statistically significant test results for the same strategies. The **bottom left panel** displays the

mean observed effect size for the same strategies. The line represents a perfect mean measurement of the true effect size. Each data point represents 15,000 simulations. Note that in the two top panels, the points for random sampling (yellow dots) and hypothesizing after a result is known (HARKing; dark blue triangles) are entirely overlapping. In the top right panel, combined QRPs (purple triangles) also overlaps with these two strategies. Similarly, in the bottom left panel, the points for random sampling and sampling with 80% power (orange dots) are entirely overlapping. QRP = questionable research practice; HARKing = hypothesizing after the result is known.

For example, if the researchers use optional stopping (green triangles in Figure S1), they sample fewer participants compared to the other strategies between r = .21 and r = .71 (top left panel). The effect on the proportion of positive results is striking, see Figure S1 top right panel. Despite the smaller sample, optional stopping leads to more positive test results in the same effect size range. However, this practice does not lead to reproducible science, given a false positive rate of 29%, compared to 5% for the two strategies avoiding questionable research practices (Figure S1 top right panel, leftmost data points represent null effect).

In addition to increasing the chance of reporting a null effect as significant, QRPs lead to effect size overestimations (Figure S1 bottom left panel). As depicted by the green triangles, a team using optional stopping observes an effect size of Pearson correlation coefficient r = .37 when in truth the population effect size is merely r = .27. If the researchers decide to combine questionable research practices (purple triangles in Figure S1), they consistently report effect sizes above r = .38 even though the true population effect size is null or small. In sum, these simulations show that the application of QRPs in original studies can explain low replication rates and lower than expected observed effect sizes in replication studies which avoid QRPs, as found empirically by the Open Science Collaboration (2015).

References

Open Science Collaboration. (2015). Estimating the reproducibility of psychological science. *Science*, *349*(6251), aac4716. http://doi.org/10.1126/science.aac4716
